# Identification of mesenchymal stem cells and osteogenic factors in bone marrow aspirate and peripheral blood for spinal fusion by flow cytometry and proteomic analysis

**DOI:** 10.1186/1749-799X-9-32

**Published:** 2014-05-03

**Authors:** Chi-Chien Niu, Song-Shu Lin, Li-Jen Yuan, Lih-Huei Chen, Tai-Long Pan, Chuen-Yung Yang, Po-Liang Lai, Wen-Jer Chen

**Affiliations:** 1 Department of Orthopaedic Surgery, Chang Gung Memorial Hospital, No 5, Fu-Hsing Street 333, Taoyuan, Kweishan, Taiwan; 2 Graduate Institutes of Biomedical Sciences, Chang Gung University, Taoyuan, Taiwan; 3 School of Traditional Chinese Medicine, Chang Gung University, Taoyuan, Taiwan; 4 Research Center for Industry of Human Ecology, Chang Gung University of Science and Technology, Taoyuan, Taiwan; 5 College of Medicine, Chang Gung University, Taoyuan, Taiwan

**Keywords:** Bone marrow aspirate, Mesenchymal stem cells, Flow cytometry, Proteomics, Lumbar posterolateral fusion

## Abstract

**Background:**

An *in vivo* animal study and a prospective clinical study have indicated that bone marrow aspirate (BMA) augments spinal arthrodesis. However, there is no quantified data to explain why fusion rate can be augmented by BMA in lumbar posterolateral fusion.

**Methods:**

To analyze the proportion of mesenchymal stem cells (MSCs) and osteogenic factors in human BMA and peripheral blood (PB) of the same patient. Autologous BMA and PB from the patients were analyzed by flow cytometry (FACS) using cell markers for MSCs. The osteogenic potential of MSCs was determined by alkaline phosphatase (ALP) activity and calcium level quantification. Proteomics were used for the qualitative and quantitative mapping of the whole proteome from BMA and PB plasma. The mass-to-charge ratio was calculated by time-of-flight mass spectrometry (TOF-MS). The overexpression of protein was confirmed using Western blot analysis.

**Results:**

The proportion of MSCs (CD34^−^/CD29^+^/CD105^+^) was higher in the BMA than that in the PB. Colony-forming cell (CFC) assays suggested that fewer colonies were formed in PB cultures than in BMA culture. There was no significant difference in the osteogenic potential of the MSCs between the PB and BMA. Proteomic mass spectrometry assays suggested that the levels of catalase (osteoclast inhibitor) and glutathione peroxidase 3 (osteogenic biomarker) were higher in the BMA than those in the PB, and this was confirmed by Western blot analysis.

**Conclusions:**

The proportions of MSCs and osteogenic factors were higher in the BMA than in the PB. This may explain why fusion rate can be augmented by BMA in lumbar posterolateral fusion.

## Background

Thus far, autologous bone graft is the gold standard for spinal fusion. Autologous cancellous bone harvested from the ilium is commonly used in intertransverse fusion of the lumbar spine. However, the incidence of complications associated with harvesting bone from the posterior iliac crest is significant. Various graft substitutes or autograft extenders have become available or are currently under development including allografts [1,2], ceramics [3,4], demineralized bone matrix (DBM) [5,6], and cultured stem cells [7]. Some of these substitutes function as osteoconductors or as osteoinductors in the process of osteogenesis.

Bone marrow aspirate (BMA) is harvested percutaneously from a cancellous-rich site such as the iliac crest, proximal tibia, or calcaneus utilizing a bone marrow needle and large-gauge syringe. Animal studies have proven that BMA exhibits osteopromotive properties that promote spinal fusion due to the presence of osteoprogenitor cells and BMPs [8,9]. Our previous prospective clinical study indicated that BMA augments spinal arthrodesis [10]. However, there is no quantified information available in the literature.

Mesenchymal stem cells (MSCs) exhibit multipotent differentiation potential, and have been shown to give rise to different mesodermal cell lineages including osteoblasts, chondroblasts, and adipocytes [11]. MSCs have to be plastic-adherent when maintained under standard culture conditions. MSCs express CD73, CD90, and CD105 and lack expression of the hematopoietic lineage markers c-kit, CD14, CD11b, CD34, CD45, CD19, CD79α, and human leukocyte antigen (HLA)-DR [12]. The aim of this study was to analyze the proportion of MSCs in human BMA. These factors in the PB of the same patient were also evaluated and compared with the BMA.

Human plasma is easily obtainable and clinically valuable [13]. Plasma components derived from tissues and organs vary in concentration by at least nine to ten orders of magnitude [14]. Two-dimensional gel electrophoresis (2-DE) is a reliable method to quickly resolve small quantities of proteins into hundreds of protein spots with definite molecular weights and pIs, and can allow for the comparison of protein intensity between samples. With the help of MALDI-TOF MS for rapid protein identification, qualitative and quantitative changes of primary gene products and post-translational modifications (PTMs) can be monitored [15,16]. For an identified osteogenic marker to be clinically useful, it should be measurable in body fluid samples.

In this study, we analyzed the proportion of MSCs, fibroblast colony-forming units (CFU-F) assay, and the osteogenic potential of MSCs and investigated the differences between human BMA and PB. Proteomic analysis was used to identify the proteins differently expressed in BMA compared to PB and the findings were confirmed by Western blotting. These findings may explain why fusion rate can be augmented by BMA in lumbar posterolateral fusion.

## Materials and methods

We harvested the bone marrow aspirate (BMA) and peripheral blood (PB) from 14 patients (64.1 ± 9.9 years old) who received total discectomy and posterior lumbar interbody fusion with cages. A study design table is shown in Table 1. The experimental protocol was approved by the Human Subjects Institutional Review Board at the Chang Gung Memorial Hospital.

**Table 1:** Study design table

**Method**	**BMA sample number**	**PB sample number**	**Patient ID**
Flow cytometry analysis	4	4	P1-P4
CFU assay	4	4	P1-P4
ALP activity measurement	3	3	P5-P7
Calcium level quantification	3	3	P5-P7
Mass spectrometric analysis	3	3	P8-P10
Western blot analysis	6	6	P9-P14

### Surgical procedures

During the operation, the patients were put in a prone position over the four-poster frame. The skin, overlying fascia, and musculature were opened to explore the posterior elements of the lumbosacral spine and the extent of the wound depending on the number of the levels of stenosis and instability was treated. After finishing the posterior lumbosacral decompression procedure, the right iliac crest close to the posterior superior iliac spine was explored. The outer cortex was exposed by subperiosteal dissection and a 3 × 3-cm hole was created by osteotomy. During bone graft harvesting, 10 ml of BMA was aspirated and collected in a heparin-rinsed syringe.

### Flow cytometry analysis for phenotyping of MSCs

Under sterile conditions, 1 ml of BMA or PB was collected into a heparin-rinsed syringe. Two ml of 1X BD Pharm Lyse™ lysing solution (Becton Dickinson, Franklin Lakes, NJ, USA) was then added to each tube containing up to 200 μl of a whole blood plus anti-CD29 FITC-conjugated, anti-CD34 RPE conjugated and anti-CD105 Alexa Fluro-conjugated Ab (AbD Serotec, Oxford, UK) mixture and incubated at room temperature, protected from light, for 15 min. Cell suspensions were washed, centrifuged, suspended, and analyzed on a FACS Calibur flow cytometer with CellQuest software (Becton Dickinson).

### Isolation of MSCs from BMA and peripheral blood

Under sterile conditions, 10 ml of BMA or PB was collected into a heparin-rinsed syringe. The sample was washed with PBS. Cells were recovered after centrifugation at 600 g for 10 min. Up to 2 × 10^8^ of nucleated cells in 5 ml of DPBS were loaded onto 25 ml of Percoll cushion (Pharmacia Biotech, Piscataway, NJ, USA) of a density of 1.073 g/ml in a 50-ml conical tube. Cell separation was accomplished by centrifugation at 1,100 g for 40 min at 20°C. The nucleated cells were collected from the interface, diluted with two volumes of PBS, and collected by centrifugation. The cells were then re-suspended, counted, and plated at 10^5^ cells in T-75 flasks (Coring, MA, USA). The cells were maintained in complete medium (DMEM-low glucose) (Gibco, Gran Island, NY, USA) containing 10% fetal bovine serum (FBS) and antibiotics (mixture of 100 units/ml of penicillin and 100 μg/ml of streptomycin) (Gibco) at 37°C in a humidified atmosphere of 5% CO_2_ and 95% air. After 4 days of primary culture, the non-adherent cells were removed by changing the medium. The medium was changed every 3 days thereafter. MSCs grow as symmetrical colonies and were subcultured at 10 to 14 days.

### Colony-forming units (CFU-F) assay

After the BMA or PB was enriched with Percoll [11], 10^7^ mononuclear cells were plated in a 100-mm dish, cultured for 2 weeks at 37°C in 5% humidified CO_2_ atmosphere, fixed with methanol at −20°C for 5 min, and finally stained with Giemsa for counting. The number of colonies was determined by image analysis of each dish with Imagine-Pro version 5 software (Media Cybernetics, Rockville, MD, USA).

### Alkaline phosphatase (ALP) activity measurement

MSCs from PB or BMA were cultured in complete medium or induction medium (consisting of complete medium supplemented with 10^−9^ M dexamethasone 20 mM β-glycerol phosphate and 50 μg/ml ascorbate-2-phosphate) to measure the activity of ALP activity. At 4, 8, 12, and 16 days after culture, the culture medium was withdrawn and the dish was washed twice with 10 ml of Tyrode's balanced salt solution. A 10-ml aliquot of ALP substrate buffer (50 mM glycine, 1 mM MgCl2, pH 10.5), containing the soluble chromogenic ALP substrate (2.5 mM *p*-nitrophenyl phosphate), was added at room temperature. During incubation, cell-surface ALP converts *p*-nitrophenyl phosphate into *p*-nitrophenol and changes to a yellowish color. Twenty minutes after addition of the substrate, 1 ml of the buffer was removed from the culture and mixed with 1 ml of 1 N NaOH to stop the reaction. The absorbance of the mixture was read in triplicate on an ELISA MRX plate reader (Dynatech Labs, USA) at 405 nm. The DNA content was determined by DNAzol reagent (Invitrogen, Carlsbad, CA, USA) according to the manufacturer's instructions. Enzyme activity was expressed as nmol *p*-nitrophenol/min/μg DNA.

### Calcium level quantification

MSCs from PB or BMA were cultured in induction medium for 21 days. The culture dishes were rinsed twice with Tyrode's balanced salt solution and then poured into a 50-ml tube containing 10 ml of 0.5 N HCl. Calcium was extracted from the cells by shaking for 24 h at 4°C. Cellular debris was centrifuged and the calcium in the supernatant was measured quantitatively by a QuantiChrom™ calcium assay kit (BioAssay Systems, Hayward, CA, USA) according to the manufacture'st instruction. Absorbance of the samples was measured on a MRX multiplate reader (Dynatech Labs, USA) at 570 nm for 5 to 10 min after the addition of the pertinent reagents. DNA content was determined by DNAzol reagent (Invitrogen) according to the manufacture's protocol description. Total calcium was calculated from standard curves of solutions prepared in parallel with the experiments and expressed as μg Ca/μg DNA.

### Two-dimensional gel electrophoresis and silver staining

Six serum samples from PB or BMA for proteomic analysis were collected. To improve the performance of the analysis of the serum samples, albumin and immunoglobulin G in the collected serum samples were depleted using an albumin and IgG removal kit (Depletion Spin Trap, GE Healthcare UK Limited, Amersham, Buckinghamshire, UK) according to the manufacturer's instructions. The procedures for 2-DE gel running, staining, and imaging were as previously described [17-19]. Briefly, aliquots in sample buffer (7 M urea, 2 M thiourea, 4.5% CHAPS, 100 mM DTE, 40 mM Tris, pH 8.8) were applied to immobilized pH 3 to 10 nonlinear gradient strips (Amersham Biosciences, Uppsala, Sweden). IEF was performed at 80,000 Vh. The second dimension was analyzed on 9% to 16% linear gradient polyacrylamide gels at constant 40 mA per gel for approximately 5 h. Proteins resolved by 2-DE were visualized by silver staining as previously described [17-19]. Briefly, the gels were fixed in 30% methanol for 15 min and then subjected to washing with water, followed by 2 min in sodium thiosulfate (0.8 mM), then washing again with water and subsequently equilibrated with 0.2% *w*/*v* silver nitrate for 25 min. The gels were then rinsed three times with water and developed in 3% sodium carbonate containing 0.04% *v*/*v* formaldehyde (37% solution). The reaction was stopped with 40 mM EDTA and the gels were washed three times with water. Finally, the gels were scanned and the images analyzed using Image Master 2D software, version 4.01.

### In-gel digestion of proteins and mass spectrometric analysis

More than 1.5-fold increased or decreased silver-stained spots were excised and in-gel digested with trypsin according to procedures described previously [17-19]. Briefly, the gels were destained by 1% potassium ferricyanide and 1.6% sodium thiosulfate (Sigma, St. Louis, MO, USA). The proteins were then reduced with 25 mM NH_4_HCO_3_ containing 10 mM DTT (Biosynth, Switzerland) at 60°C for 30 min and alkylated with 55 mM iodoacetamide (Amersham Biosciences, Amersham, Buckinghamshire, UK) at room temperature for 30 min. After reduction and alkylation, the proteins were digested with trypsin (Promega, Madison, WI, USA) (20 mg/ml) at 37°C overnight. After digestion, the tryptic peptides were acidified with 0.5% TCA and loaded onto an MTP Anchor Chip™ 600/384 TF (Bruker Daltonik GmbH, Bremen, Germany). MALDI-TOF MS analysis was performed on an Ultraflex™ MALDI-TOF mass spectrometer (Bruker Daltonik). Mono-isotopic peptide masses were assigned and used for database searches with the MASCOT search engine (http://www.matrixscience.com) (Matrix Science, London, UK).

### Western blot analysis

Twelve serum samples from PB or BMA were collected for Western blotting. The protein content was quantitated using a protein assay kit (Pierce Biotechnology, Rockford, IL, USA), separated by 6% SDS-PAGE for catalase and 10% for glutathione peroxidase and β-actin, and transferred onto membranes using a transfer unit (Bio-Rad, Hercules, CA, USA). After blocking, the membranes were incubated with 1,000-fold diluted rabbit antibodies against catalase and glutathione peroxidase (Abcam, Cambridge, UK) or mouse antibodies against β-actin (Millipore, Temecula, CA, USA). After washing, the membranes were further incubated for 2 h with 10,000-fold goat anti-mouse IgG (Calbiochem, Millipore, Billerica, MA, USA) or goat anti-rabbit IgG (Millipore) conjugated to horseradish peroxidase. The membranes were then washed and rinsed with ECL detection reagents (Amersham Pharmacia Biotech, Amersham, Buckinghamshire, UK). The band images were photographed using ECL Hyperfilm (Amersham).

## Results

### MSCs (CD34^−^/CD29^+^/CD105^+^) isolated from PB and BMA

**Figure 1:**
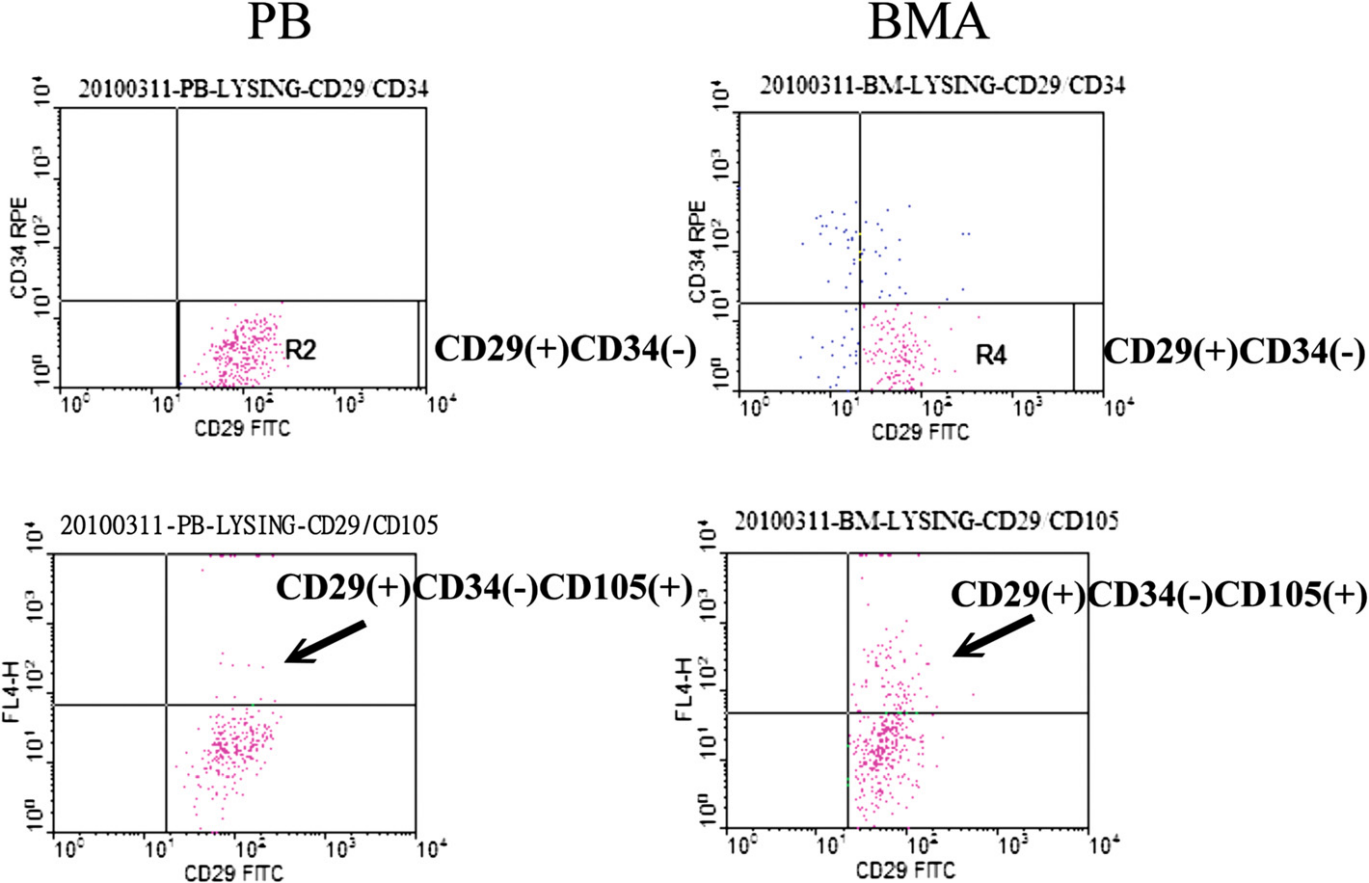
**Flow cytometry analysis of MSC-like cells from PB and BMA.** The proportion of CD34^−^/CD29^+^/CD105^+^-nucleated cells in the PB was significantly lower than that in the BMA (*t* test, *p* < 0.01, *n* = 4).

The proportion of CD34^−^/CD29^+^/CD105^+^-nucleated cells in the PB was significantly lower than that in the BMA (Figure 1, 0.06% ± 0.03% vs. 0.24% ± 0.06%, *p* < 0.01, *n* = 4).

### Colony-forming units assay

**Figure 2:**
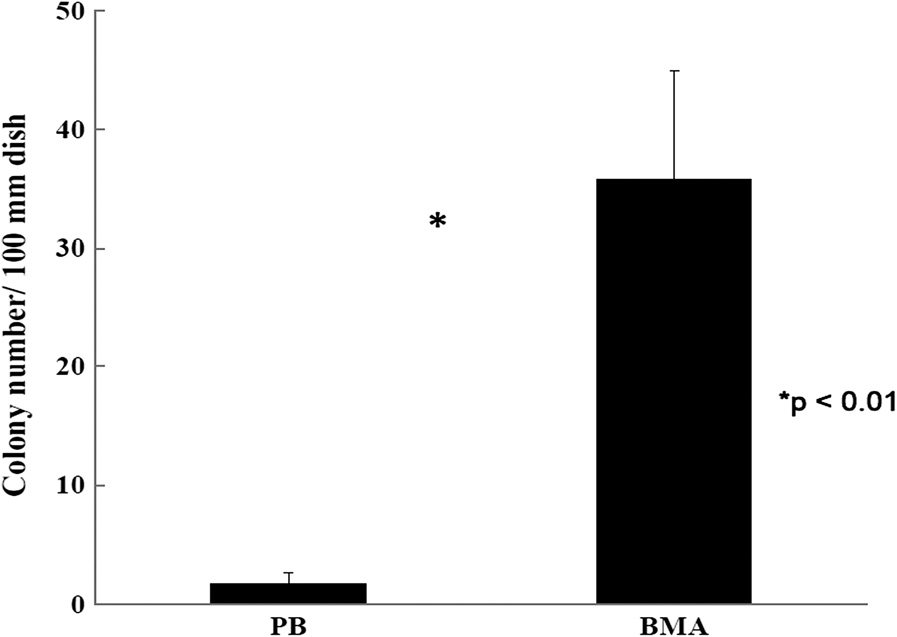
**Fibroblast-colony-forming units (CFU-F) assay.** Fewer colonies were formed in the PB cultures than in BMA cultures (*t* test, *p* < 0.01, *n* = 4).

To determine the clonogenic potential of MSCs, colony-forming units (CFU-F) assays were performed after 2 weeks of culture. The mean colony-forming efficiency was 1.8 ± 0.9 mononuclear cells for the PB and 35.8 ± 9.1 mononuclear cells for the BMA. Fewer colonies were formed in the PB cultures than in the BMA cultures (Figure 2, *p* < 0.01, *n* = 4).

### Alkaline phosphatase activity measurement

**Figure 3:**
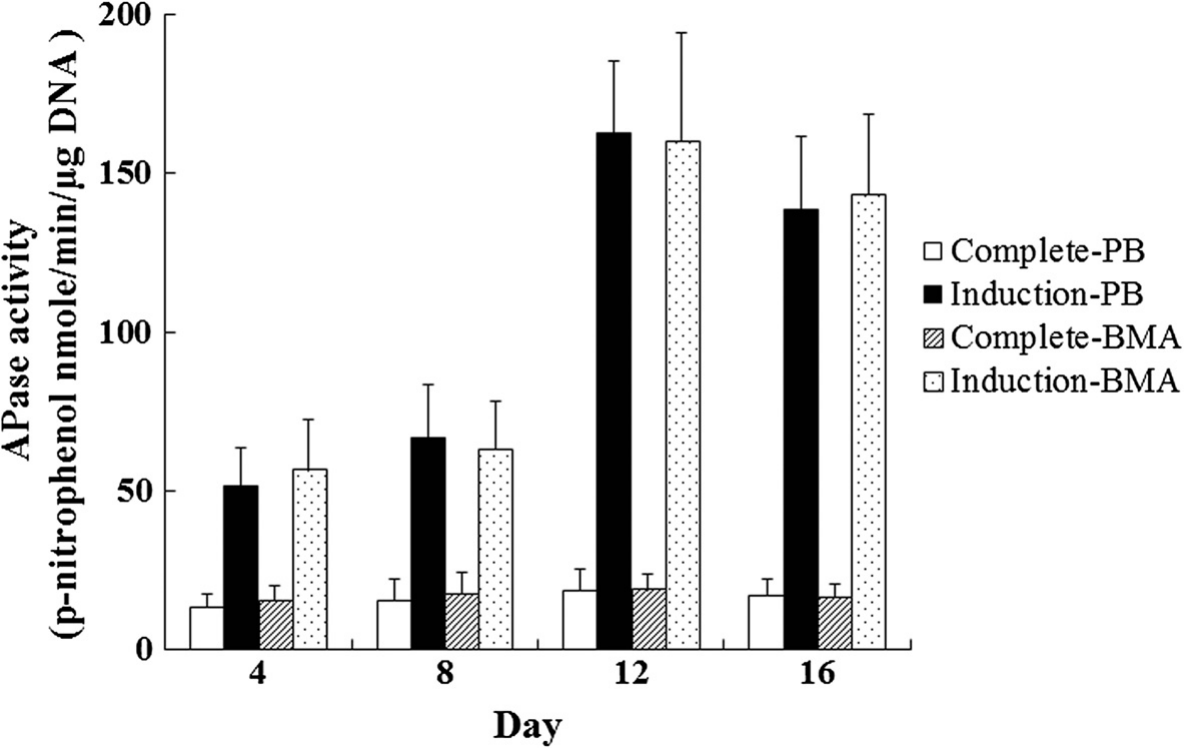
**Alkaline phosphatase (ALP) activity measurement.** There was higher ALP activity in the induction medium culture than in the complete medium culture in both PB and BMA (*t* test, *p* < 0.01, *n* = 3). There were no significant differences in ALP activity between the PB and BMA in complete medium or induction medium culture in each time point shown (*t* test, *p* > 0.05, *n* = 3).

For the PB group, alkaline phosphatase (ALP) activity measured in the complete medium was 13.2 ± 4.1, 15.1 ± 7.2, 18.3 ± 6.7, and 17.1 ± 5.1 nmol *p*-nitrophenol/min/μg DNA after 4, 8, 12, and 16 days, respectively. ALP activity measured in the induction medium was 51.5 ± 12.2, 66.5 ± 17.2, 162.7 ± 22.4, and 138.3 ± 23.5 nmol *p*-nitrophenol/min/μg DNA after 4, 8, 12, and 16 days, respectively. There was higher ALP activity in the induction medium than in complete medium culture in each time point showed (Figure 3, *p* < 0.01, *n* = 3).

For the BMA group, ALP activity measured in the complete medium was 15.2 ± 5.1, 17.2 ± 6.9, 18.6 ± 5.2, and 16.4 ± 4.3 nmol *p*-nitrophenol/min/μg DNA after 4, 8, 12, and 16 days, respectively. ALP activity measured in the induction medium was 56.5 ± 16.2, 62.9 ± 115.4, 160.1 ± 33.9, and 143.2 ± 25.2 nmol *p*-nitrophenol/min/μg DNA after 4, 8, 12, and16 days, respectively. There was higher ALP activity in the induction medium than in the complete medium culture at each time point (Figure 3, *p* < 0.01, *n* = 3). However, there was no significant difference between the PB and BMA in complete medium or induction medium culture in each time point showed (Figure 3, *p* > 0.05, *n* = 3).

### Calcium level quantification

**Figure 4:**
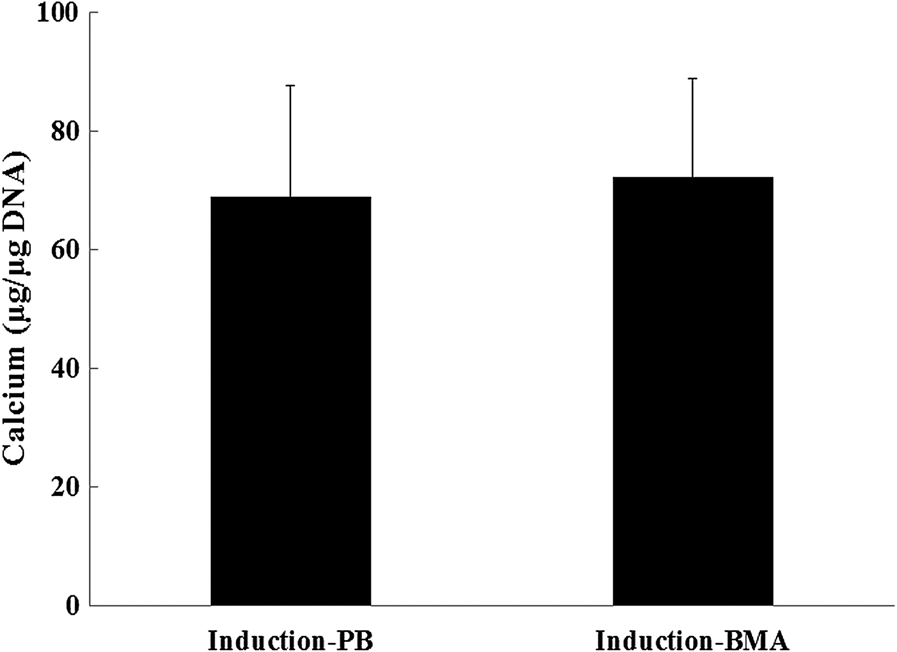
**Calcium level quantification.** There was no significant difference in calcium level between the PB and BMA (*t* test, *p* > 0.05, *n* = 3).

The calcium level was 69 ± 18.5 μg Ca/μg DNA for the PB group and 72.3 ± 16.4 μg Ca/μg DNA for the BMA group. There was no significant difference between the PB and BMA in calcium level (Figure 4, *p* > 0.05, *n* = 3).

### Protein profiles of BMA and paired PB

**Figure 5:**
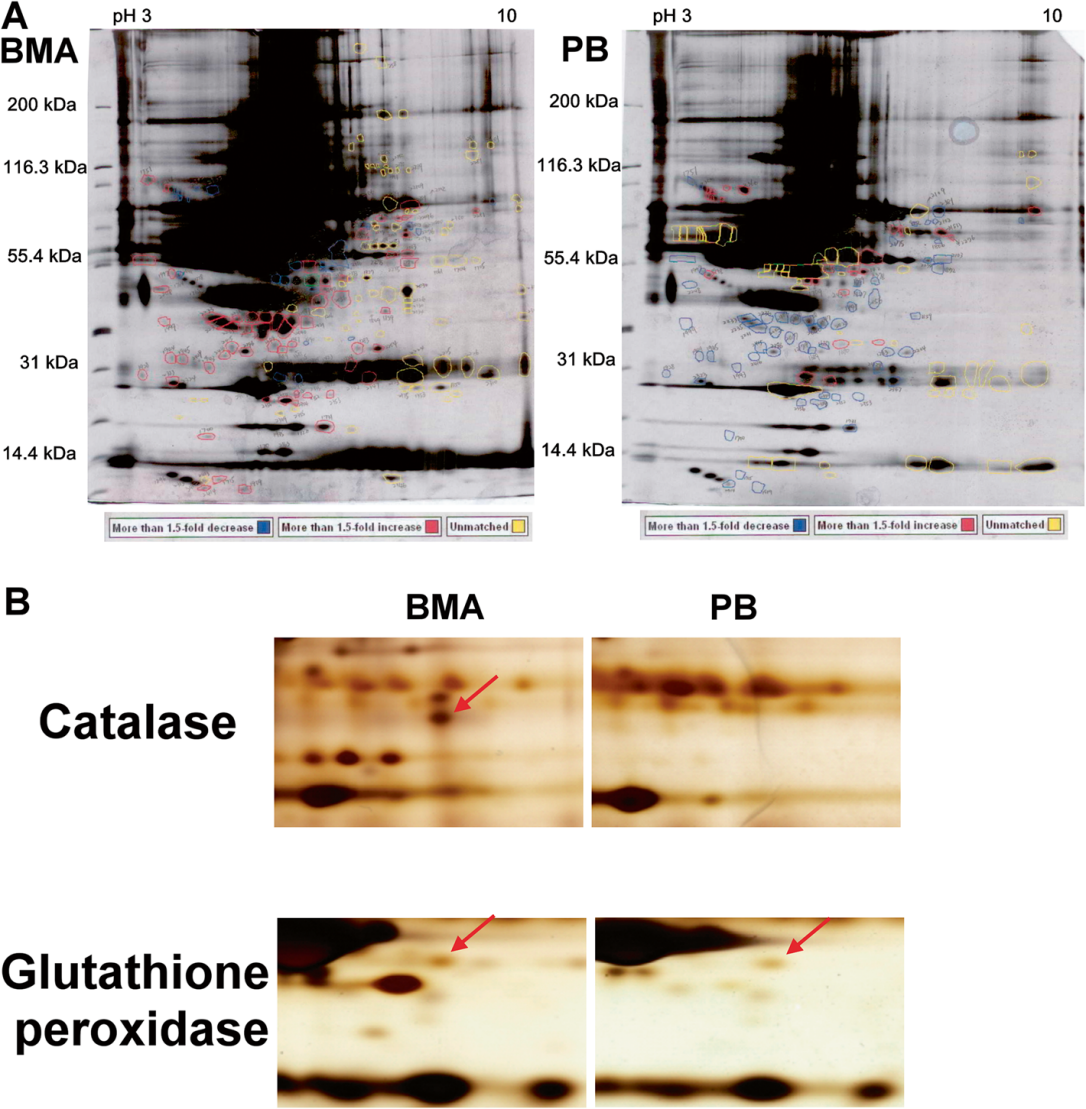
**Protein profiling of BMA and PB specimens. (A)** Proteins (150 mg) were subjected to 2-DE (pH 3 to 10) and detected by silver staining. Spots that had more than a 1.5-fold increase or decrease in silver staining were excised and in-gel digested with trypsin. At least 11 protein spots were detected at higher expression levels and 5 at lower expression levels in the BMA than in the PB. **(B)** Cropped images showing the two selected proteins (catalase and Gpx) from 2-D gels.

**Figure 6:**
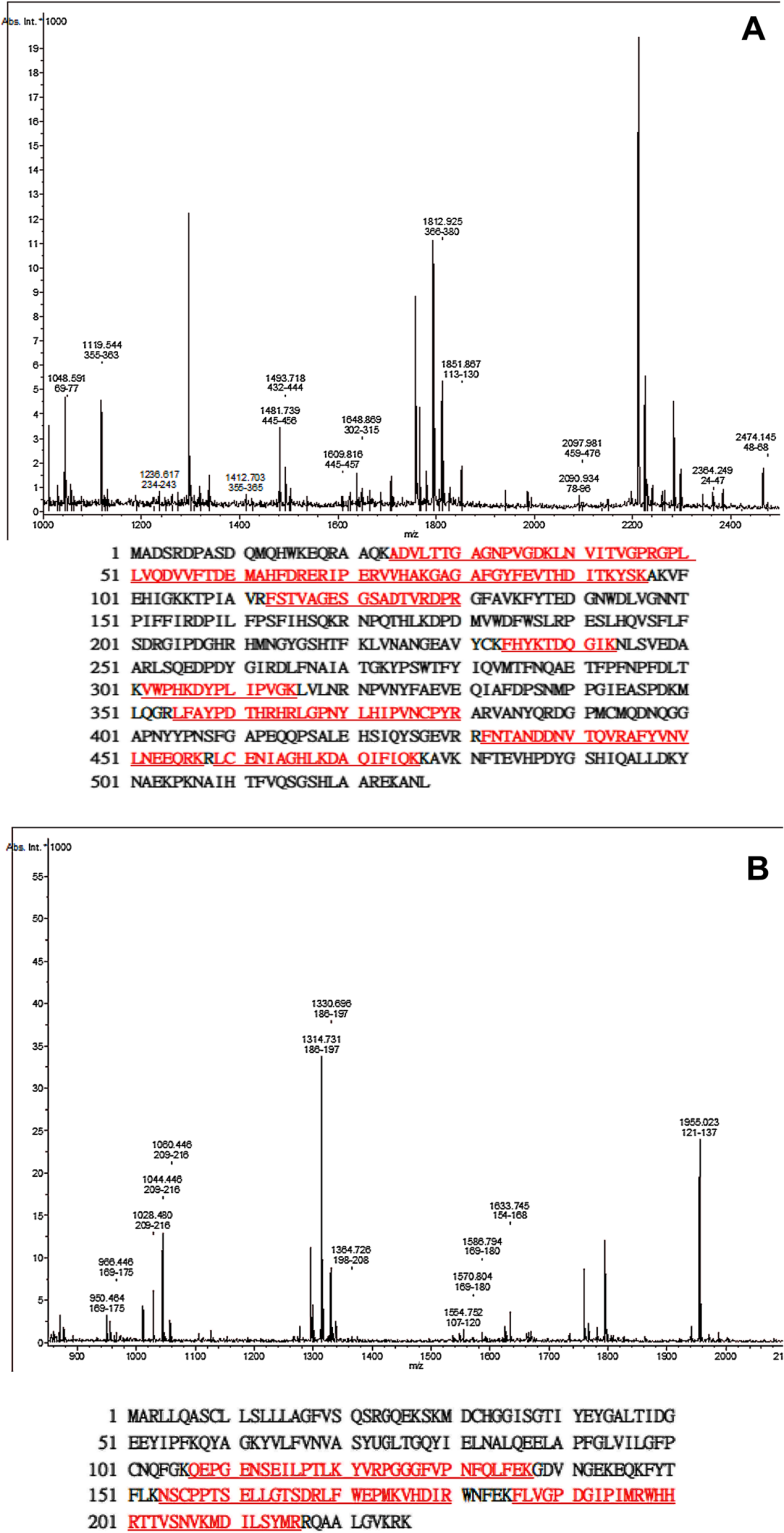
**Mass spectrometric analysis of the protein spots.** Protein spots of catalase **(A)** and Gpx **(B)** were picked, in-gel digested with trypsin, and analyzed by MALDITOF MS. The matched peptides from mass spectrometric analysis of both spots to the amino acid sequence of human catalase and Gpx are underlined and in bold.

In the proteins extracted from the paired specimens, at least 11 protein spots were detected at higher expression levels and 5 at lower expression levels in the BMA than in the PB (Figure 5a). MALDI-TOF MS was used to identify the proteins corresponding to these 16 spots, and the relative levels of these proteins between the BMA and PB were quantitated by image analysis (Table 2). Although other spots also showed different intensities between the BMA and PB in 2-DE, they could not be definitively identified by MS. We thus focused on the 16 spots that showed differences in intensity and could be unequivocally identified by MS. At least two osteogenesis-related proteins were found, and there were higher catalase (osteoclast inhibitor) and glutathione peroxidase 3 (osteogeneic biomarker) content in the BMA than in the PB (Figure 5b). The identities of these two protein spots were confirmed by MALDI-TOF MS and shown in Figure 6.

**Table 2:** 2-DE and MALDI-TOF MS identification of proteins that are differentially expressed in BMA and PB

**Spot**	**Protein name**	**Mr**	**pI**	**Score**^**a**^	**Sequence coverage**^**b **^**(%)**	**Trend**
1	Hemoglobin subunit beta	16,102	6.75	68	57	BMA > PB
2	Catalase	59,947	6.9	206	53	BMA > PB
3	Ferritin light chain	20,064	5.51	142	63	BMA > PB
4	Flavin reductase	22,219	7.13	179	89	BMA > PB
5	Protein S100-A8	10,885	6.51	103	67	BMA > PB
6	Complement C3	188,569	6.02	66	11	BMA > PB
7	Fibrinogen beta chain	56,577	8.54	148	51	BMA > PB
8	Hemopexin	52,385	6.55	121	41	BMA < PB
9	Serotransferrin	79,280	6.81	92	25	BMA < PB
10	Transthyretin	15,991	5.52	155	73	BMA > PB
11	Clusterin	53,031	5.89	108	41	BMA > PB
12	Apolipoprotein A-I	30,759	5.56	113	49	BMA < PB
13	Peroxiredoxin-2	22,049	5.66	181	72	BMA > PB
14	Leucine-rich alpha-2-glycoprotein	38,382	6.45	89	42	BMA < PB
15	Fibrinogen gamma chain	52,106	5.37	131	48	BMA < PB
16	Glutathione peroxidase	25,765	8.26	67	39	BMA > PB

^a^The MASCOT search score of identified proteins is shown; ^b^ the percentage of sequence coverage (Seq Cov) of matched peptides in the identified protein.

### Western blot analysis of proteins expression in BMA and PB

**Figure 7:**
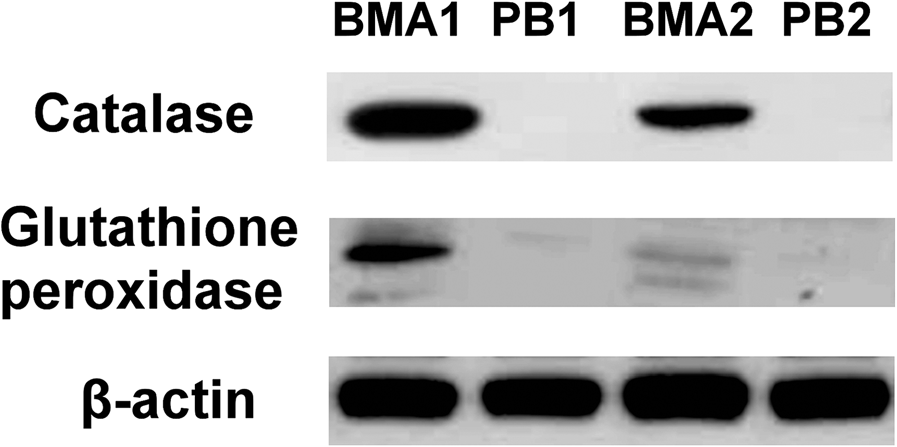
**Western blot analysis of catalase and Gpx in BMA and PB.** The protein levels of catalase and glutathione peroxidase in the BMA were significantly higher than those in the PB. The level of β-actin in each sample detected by Western blot was used as a loading control.

To confirm the data obtained from 2-DE analysis, the serum was detected by Western blotting with specific antibodies. Our results revealed that the protein levels of catalase and glutathione peroxidase in the BMA were significantly higher than those in the PB (Figure 7).

## Discussion

Morphologically, no specific characteristics distinguish MSCs from other nucleated cells within BMA or PB. To distinguish MSCs, specific markers reacting with cell receptor sites instead must be identified. Specific monoclonal antibodies react with molecules on specific cell surfaces and can be used to identify the protein clusters for the markers which can facilitate the identification, isolation, and study of stem cells. Multiple surface markers have been associated with stem cells including CD29, CD34, CD106, CD105, and CD309 (KDR) [20-22].

The use of FACS has the potential to identify MSCs more precisely. CD29 and CD105 are expressed by MSCs [11,23] whereas CD34 is expressed by hematopoietic stem cells and capillary endothelial progenitor cells but not MSCs [11,20,23]. In the present study, the results suggested that the proportion of MSC-like cells (CD34^−^/CD29^+^/CD105^+^) in the PB was significantly lower than that in the BMA, which provides quantified information for the osteopromotive effects of BMA (Figure 1). In addition, CFU-F assays also suggested that fewer colonies were formed in the PB cultures than in BMA cultures (Figure 2). CFU-Fs are clearly indicative of cells capable of forming colonies and are representative of the more highly proliferative cells in these cultures [21]. Under proper experimental conditions, BMA CFU-Fs are able to differentiate into bone, cartilage, adipocytes, fibrous tissues, and hematopoietic supporting tissues *in vitro* and *in vivo*[11,22]. In the present study, the osteogenic potential of CFU-Fs was determined by ALP activity (Figure 3) and calcium level (Figure 4), and no significant differences between the PB and BMA were found. Although, fewer colonies were formed in the PB cultures than in BMA cultures (Figure 2), the CFU-Fs isolated from these two groups had similar proliferation and differentiation abilities.

Connolly et al. [24] clinically found the osteopromotive effects of whole bone marrow when autologous marrow was injected into a tibial defect site. Although both blood and bone marrow can be immediately harvested and used as autologous materials for osteopromotion, an *in vivo* animal study [9] showed that BMA has a higher osteogenic potential than blood when it is impregnated through β-tricalcium phosphate (β-TCP), an osteoconductive scaffold. The authors have previously reported on the use of BMA in combination with the scaffold material at grafting sites and indicated that BMA augments spinal arthrodesis [10]. The reason for this difference may be the proportion of bone marrow MSCs in BMA.

To find possible factors to gain new insight into the mechanisms of bone formation and development, we subjected proteins extracted from BMA specimens and paired PB to 2-DE separation, silver staining, and MALDI-TOF MS analysis (Figures 5 and 6). At least two osteogenesis related proteins were found: there were higher catalase and glutathione peroxidase 3 (Gpx 3) content in the BMA than in the PB. The protein level of catalase was significantly up-regulated in a time-dependent manner during osteogenic differentiation thus reduce the excess reactive oxygen species (ROS) production to hamper the osteogenic differentiation of MSCs [25]. In addition, catalase has been reported to partially inhibit ascorbic acid-induced osteoclastogenesis [26]. Selenite enhanced the gene expression and activity of Gpx, reversed the decreased total antioxidant capacity and reduced glutathione, and against hydrogen peroxide-induced inhibition of osteoblastic differentiation of MSCs [27].

Aspiration of autogenous bone marrow cells from the iliac crest is a simple and safe procedure that can be performed with the patient under anesthesia and involves little to no donor site morbidity. The use of such material at bone-grafting sites has the potential to produce a significant quantity of viable bone [10,28]. BMA may have the ability to facilitate osteogenesis by acting as both an osteoprogenitor and osteoinductor to supplement the limited amount of available autologous bone for multilevel spinal fusion.

In the present study, the proportions of MSCs and osteogenic factors were higher in the BMA than in the PB. This may explain why fusion rate can be augmented by BMA in lumbar posterolateral fusion. In the future, we will evaluate whether the fusion rate of autogenous laminectomy bone chips could be augmented by BMA in a rabbit model.

## Conclusion

The proportions of MSCs and osteogenic factors were higher in the BMA than those in the PB. This may explain why fusion rate can be augmented by BMA in lumbar posterolateral fusion.

## Competing interests

The authors declare that they have no competing interests.

## Authors' contributions

NCC, LSS, and CWJ did the study design. YLJ and YCY analyzed and interpreted the data. NCC, CYS, and CWJ provided the study material or patients. PTL, LPL, and CLH drafted the manuscript. All authors read and approved the final version of the manuscript.
